# Patterned PVA Hydrogels with 3D Petri Dish^®^ Micro-Molds of Varying Topography for Spheroid Formation of HeLa Cancer Cells: In Vitro Assessment

**DOI:** 10.3390/gels10080518

**Published:** 2024-08-06

**Authors:** Maira Moreno Valtierra, Adriana Urue Corral, Jorge Armando Jiménez-Avalos, Erika Barbosa Avalos, Judith Dávila-Rodríguez, Norma Morales Hernández, Mauricio Comas-García, Guillermo Toriz González, Antonio Oceguera-Villanueva, José Alfonso Cruz-Ramos, Rodolfo Hernández Gutiérrez, Moisés Martínez Velázquez, Zaira Yunuen García Carvajal

**Affiliations:** 1 Biotecnología Médica y Farmacéutica, Centro de Investigación y Asistencia en Tecnología y Diseño del Estado de Jalisco (CIATEJ), Av. Normalistas # 800, Col. Colinas de la Normal, Guadalajara 44270, Mexico; vmaira86@hotmail.com (M.M.V.); adriana.urue29@gmail.com (A.U.C.); avalos.joar@gmail.com (J.A.J.-A.); 2 Centro de Investigación y Desarrollo Oncológico, S.A. de C.V. (CIDO), Av. Palmira # 600-A, Col. Villas del Pedregal, San Luis Potosí 78218, Mexico; 3 Facultad de Ciencias, Universidad Autónoma de San Luis Potosí, Av. Parque Chapultepec # 1570, San Luis Potosí 78210, Mexico; mauricio.comas@uaslp.mx; 4 Laboratorio de Anatomía Patológica, Hospital Civil Viejo Fray Antonio Alcalde, Coronel Calderón #777, El Retiro, Guadalajara 44280, Mexico; dra_barbosa@hotmail.com (E.B.A.); jrdaguez@gmail.com (J.D.-R.); 5 Tecnología Alimentaria, Centro de Investigación y Asistencia en Tecnología y Diseño del Estado de Jalisco (CIATEJ), Camino Arenero # 1227, Col. El Bajío del Arenal, Zapopan 45019, Mexico; nmorales@ciatej.mx; 6 Centro de Investigación en Ciencias de la Salud y Biomedicina, Universidad Autónoma de San Luis Potosí, Sierra Leona # 550 Lomas de San Luis, San Luis Potosí 78210, Mexico; 7 Departamento de Madera, Celulosa y Papel, Centro Universitario de Ciencias Exactas e Ingenierías, Universidad de Guadalajara, Carretera Guadalajara-Nogales km 15.5, Zapopan 45220, Mexico; torizgmo@gmail.com; 8 Instituto Jalisciense de Cancerología, Secretaría de Salud Jalisco, 715 Coronel Calderón St., El Retiro, Guadalajara 44280, Mexico; ocegueraantonio@hotmail.com (A.O.-V.); jalfonso.cruz@academicos.udg.mx (J.A.C.-R.)

**Keywords:** 3D cell culture, cell spheroids, cell aggregates, PVA hydrogel

## Abstract

Cell spheroids are an important three-dimensional (3D) model for in vitro testing and are gaining interest for their use in clinical applications. More natural 3D cell culture environments that support cell–cell interactions have been created for cancer drug discovery and therapy applications, such as the scaffold-free 3D Petri Dish^®^ technology. This technology uses reusable and autoclavable silicone micro-molds with different topographies, and it conventionally uses gelled agarose for hydrogel formation to preserve the topography of the selected micro-mold. The present study investigated the feasibility of using a patterned Poly(vinyl alcohol) hydrogel using the circular topography 12–81 (9 × 9 wells) micro-mold to form HeLa cancer cell spheroids and compare them with the formed spheroids using agarose hydrogels. PVA hydrogels showed a slightly softer, springier, and stickier texture than agarose hydrogels. After preparation, Fourier transform infrared (FTIR) spectra showed chemical interactions through hydrogen bonding in the PVA and agarose hydrogels. Both types of hydrogels favor the formation of large HeLa spheroids with an average diameter of around 700–800 µm after 72 h. However, the PVA spheroids are more compact than those from agarose, suggesting a potential influence of micro-mold surface chemistry on cell behavior and spheroid formation. This was additionally confirmed by evaluating the spheroid size, morphology, integrity, as well as E-cadherin and Ki67 expression. The results suggest that PVA promotes stronger cell-to-cell interactions in the spheroids. Even the integrity of PVA spheroids was maintained after exposure to the drug cisplatin. In conclusion, the patterned PVA hydrogels were successfully prepared using the 3D Petri Dish^®^ micro-molds, and they could be used as suitable platforms for studying cell–cell interactions in cancer drug therapy.

## 1. Introduction

The name “cancer” is designated to a broad group of diseases that can affect any part of the body [1]. Cancer is one of the most severe pathologies that causes one in six deaths worldwide [2]. Although considerable efforts have been made to develop effective drug therapy approaches to cure cancer, the exorbitant costs and the sluggish pace of new drug discovery and development have led to high failure rates [3].

Cancer drug efficacy assessments usually use animal models. However, cellular platforms provide cost-effective information on cellular responses to drugs [4]. Two-dimensional (2D) platforms with cultured flat monolayer cells continue to be the most used. In this case, cell cultures are exposed to drug candidates, usually at constant concentrations, for a fixed period to select and classify test drugs to evaluate whether they have an anticancer effect, usually quantified and measuring cell viability [4,5]. However, various drawbacks and limitations are still of concern. They cannot fully reproduce the properties of in vivo solid tumors; that is to say, the three-dimensional (3D) environment in which cancer cells reside in vivo is not accurately mimicked [4,5].

Three-dimensional in vitro cancer models are a promising method for narrowing the gap between in vitro two-dimensional cell cultures and animal models due to their ability to mimic tumors in vivo more closely [4]. Hydrogels have the potential to be used to support the cultivation of cancer cells for spheroid growth as in vitro models, including growing cancer cells within porous scaffolds [4,5,6,7].

Three-dimensional cell cultures promote cells to form small sphere-shaped aggregates called spheroids [5]. Researchers often use spheroids to screen compounds in high-throughput drug development and toxicology studies [8]. Standard 3D experimental models can be split into two different methods: (1) scaffold-based and (2) scaffold-free methods [8].

In scaffold-based systems, cells are seeded in natural or synthetic materials, allowing for cell proliferation, aggregation, and 3D organization [8]. There are several methods used to form scaffold-free spheroids [6]. In the scaffold-free method, cell spheroids could be formed when cells are cultured on non-adhesive surfaces for several days. This method allows cells to interact with their substrates, other cells directly, and the extracellular matrix (ECM) in all directions [4,5,7,8].

There exists technology marketed and distributed by Sigma-Aldrich called 3D Petri Dish^®^ (www.microtissues.com). This technology enables a 3D cell culture environment without scaffolds, maximizing cell interactions and allowing for spheroid formation. The technology is based on autoclavable and reusable silicon micro-molds with small recesses and different topographies. Micro-molds are used to mold 3D Petri^®^ dish gelled agarose which fits into standard culture plates [9,10,11]. The homogenous cell suspension is sunk and seeded in the agarose hydrogel, favoring self-segregation without any influence from the ECM until spheroids are formed. Moreover, some advantages include the large number of spheroids formed per plate, the possibility of controlling the size of the spheroids, ease of maintenance, and agarose sterilization prior to the gelation process [6,10].

Poly(vinyl alcohol) or PVA is used as a coating for cell culture plates because it does not favor cell adhesion due to its low protein adsorption [12], which is related to cell surface proteins that mediate the interaction between cells or between cells and the ECM [13]. Some researchers have used PVA as a substrate for spheroid formation. Glioma spheroids formed using a PVA hydrogel as a plate coating showed irregular-sized spheroids, demonstrating that PVA-suppressed cell–substrate adhesion favors cell–cell adhesion [12]. PVA hydrogels structured with inverted pyramid-shaped microwells were used to guide the aggregation of cells into multicellular cell spheroids, which reached sizes of 157 ± 25 μm after 5 days of culture [14]. PVA membranes were seeded with human bone marrow mesenchymal stem cells (MSCs). The MSCs were self-organized into cell spheroids with an average size of between 20 and 30 μm [15]. Porous PVA/gelatin hydrogels loaded with galactose prepared by freeze/thaw cycling were used for hepatocyte cultures, obtaining cell spheroids after 7 days of cell culture [16]. Well-culture plates treated with polydimethylsiloxane and PVA were employed to form tumor spheroids with uniform sizes (0.3–1 mm) [17].

However, the 3D Petri Dish^®^ technology only uses agarose as a substrate. Therefore, we wanted to evaluate the capacity of patterned PVA hydrogels prepared with the micro-mold Petri Dish^®^ to form spheroids and determine if it impacts their size and shape. To the best of our knowledge, this is the first study in which circular topography-patterned PVA hydrogels from Petri Dish micro-molds are used to promote the self-assembly of HeLa cells. The present study aimed to fabricate patterned agarose and PVA hydrogels using 3D Petri Dish^®^ micro-molds with a circular topography (12–81) to act as a platform for the formation of HeLa spheroids using the scaffold-free technique, trying to mimic the 3D cellular microenvironment. HeLa cells were chosen because they are the most well-known and widely used in the biological research community [18].

## 2. Results and Discussion

Hydrogels as 3D models allow for the exploration of a wide range of variables that affect tumor growth, invasion, and metastasis, including the scaffold-based and scaffold-free approaches [4,5,7,8]. In particular, hydrogels developed for this application require physicochemical and biological characterization due to their biological relevance.

The 3D Petri Dish Microtissues^®^ technology is a platform that has been validated and characterized for spheroid formation. This technology is based on molds with different topographies, and it forms a hydrogel using agarose as a model polymer [9,10,11]. However, other polymers have not been evaluated using the molds of this commercially available technology.

**Figure 1:**
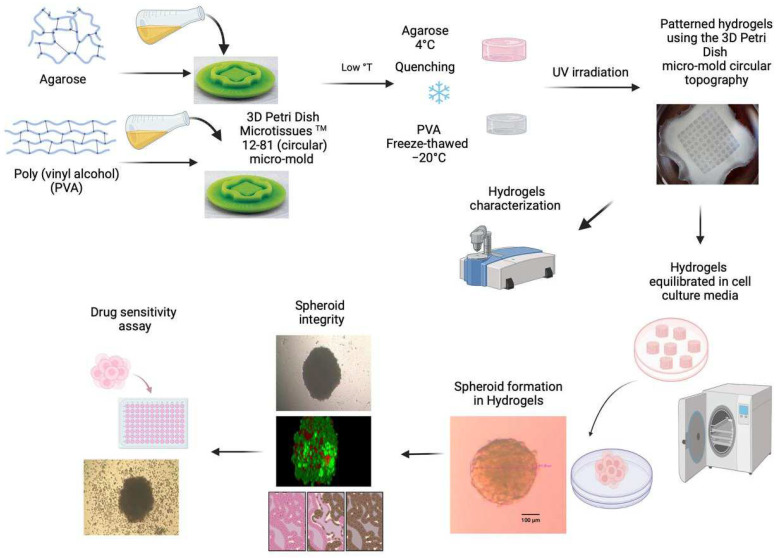
A schematic representation of hydrogel formation and their physicochemical and biological characterization.

In this study, we aimed to fabricate patterned agarose and PVA hydrogels using 3D Petri Dish^®^ micro-molds with a circular topography (12–81) to act as a platform for HeLa spheroid formation. The protocol followed for preparing the hydrogels and their biological evaluation is outlined in Figure 1.

**Figure 2:**
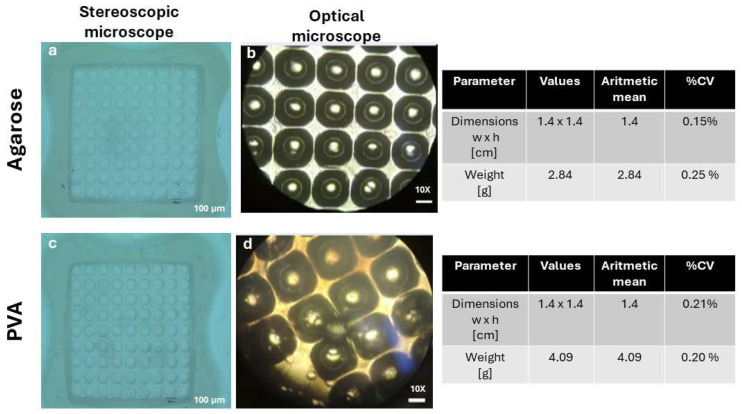
The hydrogels’ microscopic morphology, dimensions, and weight. The 3D Petri Dish Microtissues^®^ were used with a circular topography for 9 × 9 wells, identified as 12–81 micro-molds. Each of their wells has a concave bottom 800 μm long. The morphology of the obtained micro-molds was analyzed using a stereoscopic microscope (**a**,**c**). The internal morphology (pits) was analyzed using an inverted microscope (**b**,**d**).

Agarose and PVA were used due to their biocompatibility, softness, and non-adherence properties. Cell-cell interactions are favored by inducing the formation of cell aggregates that will give rise to spheroids [7,12]. Figure 2a–c shows the morphology of agarose and PVA wet hydrogels and their dimensions and weights. The 3D Petri Dish Microtissues^®^ were used with a circular topography for 9 × 9 wells, identified as 12–81 micro-molds. Each of their wells has a concave bottom 800 μm long. The morphology of the obtained micro-molds was analyzed using a microscope, and the internal morphology (pits) was analyzed in an inverted microscope.

The agarose hydrogels were manufactured following the protocol recommended by the 3D Petri Dish Microtissues^®^ technique using agarose gelation. Meanwhile, the cryogenic process with one cycle of freezing/thawing at −80 °C was used to prepare the PVA hydrogels. PVA is a hydrophilic and biocompatible polymer that can be physically cross-linked to form a hydrogel using a freeze–thaw cycle process. The obtained hydrogel possesses unique mechanical properties that can be tuned to support spheroid formation [19,20]. It is an attractive candidate to use in conjunction with 3D Petri Dish Microtissues^®^ technology.

The agarose hydrogel appearance was opaque; meanwhile, the PVA hydrogel was whitish. Edges and wells with circular geometries could be observed in the internal area. It was possible to obtain 81 uniform circular cavities with sizes of 100 μm for both hydrogels. The dimensions of the hydrogels were 1.4 cm wide and 1.4 cm long (PVA and agarose hydrogels). The hydrogels had an initial wet weight of 2.84 g for agarose and 4.404 g for PVA.

### 2.1. Swelling Behavior

The swelling properties of the agarose and PVA hydrogels were investigated in DMEM, high glucose (4500 mg/L glucose, L-glutamine, and sodium bicarbonate, without sodium pyruvate) over time. The swelling percentage determines the water-absorbing ability of hydrogels in hydrophilic physiological conditions [21].

Agarose [21,22,23,24] and PVA [25,26,27] hydrogels have a variable swelling rate depending on the pH of the aqueous medium. Hydrogels with different swelling behaviors have distinct physical properties. Swelling ability is beneficial for absorbing blood and body fluid, transferring nutrients and metabolites, and diffusing and releasing drugs in 3D cell cultures [28].

**Figure 3:**
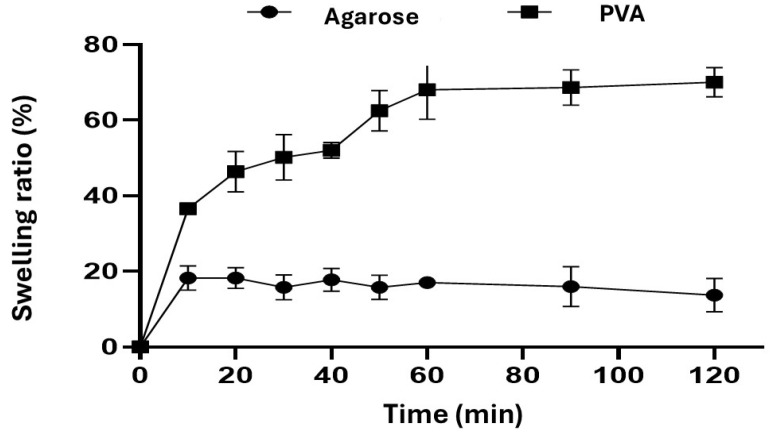
Swelling ratio (%) of hydrogels in DMEM high-glucose medium with 4500 mg/L glucose, L-glutamine, and sodium bicarbonate, without sodium pyruvate, pH~7.4 at 37 °C. Error bars signify standard deviation (SD) observed for three independent experiments.

Figure 3 shows the time-dependent swelling behavior of the agarose hydrogel prepared at cold temperatures for agarose gelling and the PVA hydrogels prepared by the freeze–thaw process. Similar swelling behavior was observed for both samples. The PVA hydrogels showed a higher swelling rate until they reached equilibrium. However, the agarose hydrogels reached a maximum swelling percentage of 20 ± 0.1% at 20 min, while the PVA hydrogels achieved it after 60 min, with 70% ± 0.1% maximum absorption. Furthermore, the agarose hydrogels revealed little swelling over time as a result of the hydrogel matrix’s degradation (at 120 min) due to stiff agarose chains [24]. It is well known that PVA hydrogels prepared via the freeze/thawing method using different freezing temperatures and drying conditions influence the swelling behavior due to the formation of hydrogen bonds among PVA molecules [25,26].

### 2.2. FTIR Analysis

**Figure 4:**
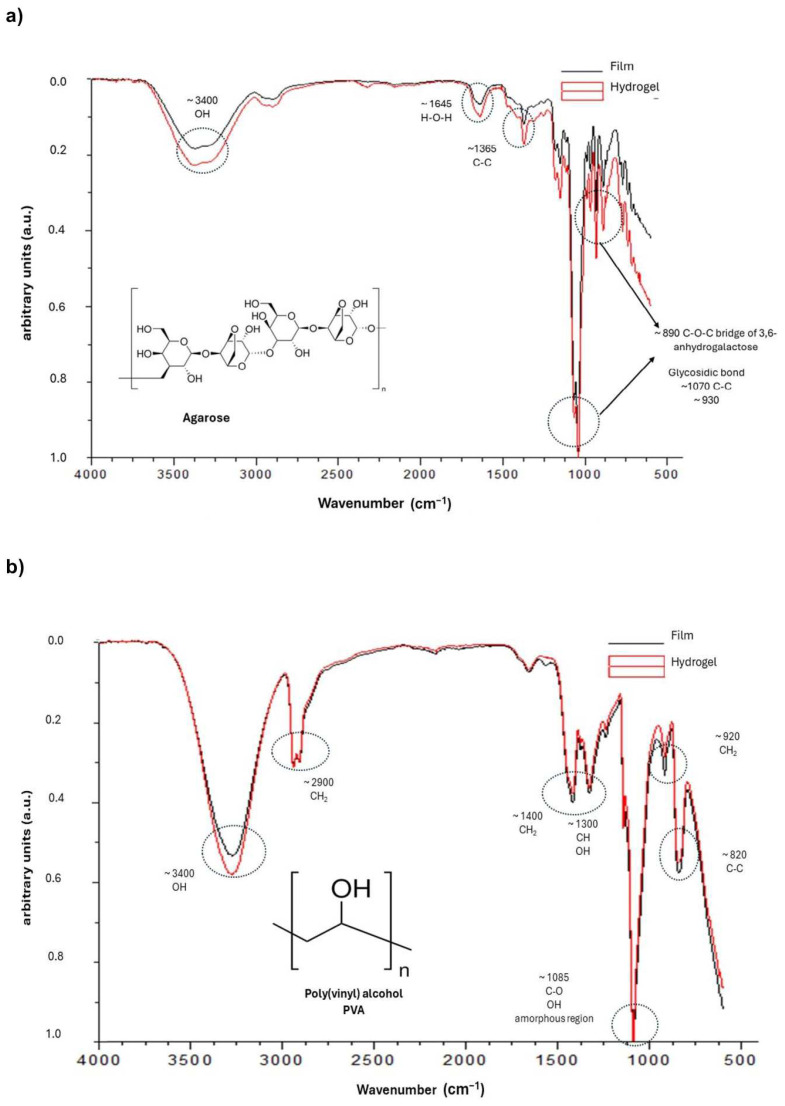
The FTIR spectrum of the agarose film and hydrogel (**a**) and the PVA film and hydrogel (**b**). The polymer chemical formula images were downloaded from the Sigma-Aldrich web page.

The ATR FT-IR spectra of the agarose and PVA hydrogels were recorded and depicted in Figure 4. Films were prepared, analyzed, and compared to the hydrogel spectra to better understand the chemical interactions. The IR spectra of agarose films and hydrogels are shown in Figure 4a. The main characteristic absorption bands of agarose were detected at 3430 cm^−1^ (stretching band of hydroxyl group), 1365 cm^−1^ (C-C), 1070 and 930 cm^−1^ (glycosidic bond), and 890 cm^−1^ (vibration of C-O-C bridge of 3,6-anhydrogalactose unit) [21,22,23,24]. The gelation of agarose is due to the aggregation of polymer chains, promoting the formation of hydrogels [29]. The FTIR peaks of the agarose hydrogels were similar to those of the films, which suggests that no covalent interactions occurred during the preparation process of the agarose hydrogels, probably due to the formation of van der Waals and/or hydrogen bonds which form a hydrogel network after physical gelation induced by a thermal process such as solvent evaporation (film) and quenching (hydrogel) [21,22,23,30,31,32].

The IR spectrum of PVA films and hydrogels (Figure 4b) showed strong peaks at 3300 cm^−1^ (OH), 2900 cm^−1^ (CH_2_), and 1085 cm^−1^ (C–O stretching and O–H bending, and the amorphous sequence of PVA). We observed a medium intensity at 1400 cm^−1^ (CH_2_ bend), 1350 cm^−1^ (scissoring O–H, rocking with C–H, wagging), 915 cm^−1^ (CH_2_ rocking), and 820 cm^−1^ (C–C stretching) [27,33,34]. The FTIR peaks of the PVA hydrogels were similar to those of the films, which suggests that no covalent interactions occurred during the preparation process of the hydrogels. Our findings agree with Mansur et al. (2008), who showed that non-covalent interactions, more specifically, intermolecular and intramolecular hydrogen bonds, are formed during the formation of films by solvent casting [34]. PVA hydrogels prepared based on physical cross-linking, such as the freezing/thawing process, favor the interaction of hydrogen bonds [19,35], stereo complex formation [36], electrostatic interaction [36], hydrophobic association [35], segment entanglement [19,20,35,36], and other non-covalent interactions [35,36] that contribute to the formation of strong hydrogen bonds in the hydrogel which mechanically reinforce the PVA network [19,20,35].

### 2.3. Texture Profile Analysis

**Figure 5:**
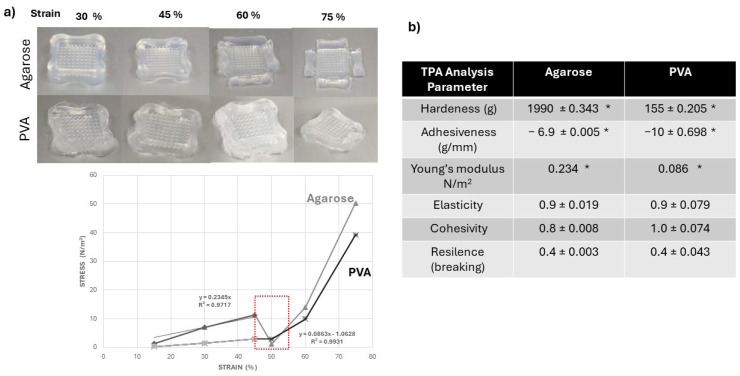
Texture profile analyses (TPAs) of the agarose and PVA hydrogels. (**a**) Detailed stages of the cyclic compression experiment and the stress vs. strain graph of the cyclic compression of the samples (**b**). The hardness, adhesiveness, and young modulus values of the agarose and PVA hydrogels presented statistical differences, The values are expressed as the mean ± standard deviation (SD) of three independent experiments. The data were analyzed with a two-way ANOVA followed by Tukey’s multiple comparison test. * *p* ≤ 0.05 comparing agarose and PVA hydrogels.

The mechanical properties of the agarose and PVA hydrogels were determined by using the texture profile analysis (TPA) method (Figure 5). The TPA is a useful tool for studying the specific mechanical properties of hydrogels as a support for cell growth [37,38,39,40].

All of the samples were punctured for a certain distance under strain, and the responsive force was measured, which can be used to examine the gel strength and elasticity of the hydrogels. This was carried out by performing a deformation test in which the strain was gradually increased by 15% at each deformation cycle until the final strain was set at 75%. The force-deformation curves (force-displacement) shown in Figure 5a represent the resistance of the sample to deformation and indicate the elastic properties (Young’s modulus, 0.23 N/m^2^ for agarose and 0.0863 N/m^2^ for PVA) resulting from the slope of the curves in the linear range at the beginning of deformation; with an increase in the strain, the responsive force also increases. This behavior is observed for both types of hydrogels. The agarose hydrogels were deformed at 50% of the strain; meanwhile, the PVA hydrogels conserved their structure at the same strain value.

Compression (hydrogel strength), hardness, adhesiveness, elasticity, cohesiveness, and resilience (breakage) were analyzed in this work in order to gain more specific knowledge about hydrogels (Figure 5b). Adhesiveness is the adhesive power of the hydrogel [41]. Cohesiveness can be measured as the degree to which materials are destroyed mechanically [41]. Resilience measures the degree of recovery of sample deformation in terms of speed and strength [41]. The agarose hydrogels exhibited values of hardness of 1990 ± 0.343 g, adhesiveness values of −6.9 ± 0.088 g/s, elasticity values of 0.9 ± 0.019, cohesiveness values of 0.8 ± 0.008, and resilience values of 0.40 ± 0.003. The agarose hydrogels were stiffer and more rigid than the PVA hydrogels.

The PVA hydrogels showed lower values for hardness (155 ± 0.205 g) and adhesiveness (−10 ± 0.69 g/s) compared to the agarose hydrogels. However, the PVA hydrogels were more elastic (0.9 ± 0.079) and cohesive (1.0 ± 0.074) than the agarose hydrogels. The values of resilience (0.44 ± 0.003) were similar between the samples of hydrogels.

Hydrogels with a high hardness are considered to have higher structural integrity, while low-hardness hydrogels can destroy structures over time [38,40]. The hardness (stiffness) and elastic properties determine the structural integrity of the hydrogel and its ability to provide mechanical support to cells and tissues [39,42,43].

Stiffness is defined as the degree to which a material resists deformation in response to an applied force [44]. The influence of stiffness on cell spreading in 3D cell cultures significantly differs from that in 2D cell cultures [42,44]. Stiffer hydrogels can provide structural support for cell attachment and proliferation [39]. Structurally stiffer and harder polysaccharide/aloe vera-based hydrogels were useful for forming tumor spheroids, suggesting that the texture of the hydrogels could influence oxygen diffusion and cell mobility during cell aggregation [40]. High adhesiveness generally increases the stickiness of the formulation, and high cohesiveness implies a good ability for structural reformation after the application, which affects hydrogel performance [45].

When the agarose gels are prepared by gelation and temperature treatment at concentrations between 0.5 and 2.0 *w*/*w*, the force required to deform them increases due to the formation of a dense cross-linking network that favors the aggregation of the polymer chains, resulting in a gel with more binding areas, thereby increasing elasticity [46].

The PVA hydrogels were prepared by using the freezing–thawing method, favoring the physical cross-links (crystallization of PVA and H bonding) between PVA chains, the strength of cross-linking density, and the interaction modes of molecular segments in the hydrogels [25,34,35]. The effect of water absorption on the mechanical properties of the PVA hydrogels is noted in the adhesive and elastic properties. It is well known that a PVA hydrogel can absorb a high-water content, modifying its mechanical properties [27].

### 2.4. In Vitro Spheroid Assessment

#### Spheroid Formation

Several methods exist to form scaffold-free spheroids [47,48,49]. In the 3D Petri Dish Microtissues^®^ technology, non-adhesive agarose hydrogels are used as micro-molded platforms with small recesses where cells settle. Micro-molded agarose guides spontaneous cell self-assembly, and cell aggregation is favored to form cellular clusters, spheroids [47], and multicellular microtissues [10,47,50,51].

The 3D Petri Dish Microtissues^®^ technology has been successfully employed for spheroid formation. Spheroid size is directly proportional to the number of cells seeded, with more cells resulting in larger spheroids. When the 12–81 mold is used and cells are seeded in a suspension (648,000 cells/190 μL), an estimated spheroid size of ~400 μm is obtained according to the microtissue protocols [9,10,11]. For example, dental pulp stem cells (DPSCs) prevascularized by human umbilical vein endothelial cells (HUVECs) were cultured and seeded in agarose gels for pulp regeneration using the molds (12–81) of the Petri Dish technology to promote the formation of spheroids, obtaining spheroids of 300 µm in diameter [51].

**Figure 6:**
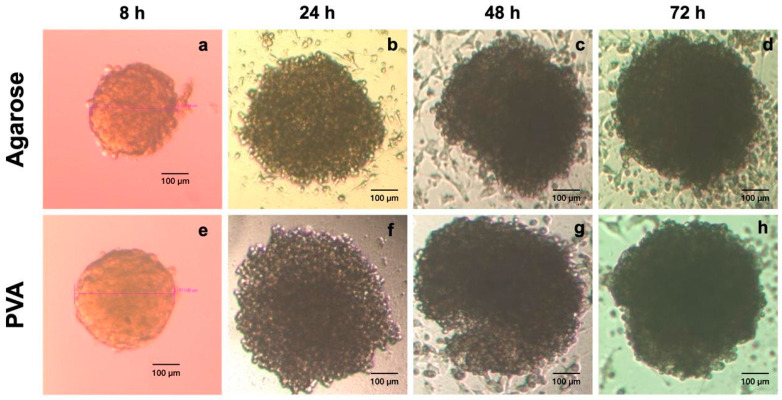
HeLa spheroid formation at different times. The monitoring of spheroid formation after 8, 24, 48, and 72 h of culturing in a standard medium in agarose hydrogels (**a**–**d**) and PVA hydrogels (**e**–**h**) with an increase in average size from ~300 to ~800 µm over time, with an initial cell density of 648,000 cells/hydrogel observed under an inverted microscope (scale bar, 100 μm).

Our findings are according to the Petri Dish protocol at the initial times. However, after 24 h, the spheroids increased in size. In the agarose and PVA hydrogels, the cells formed cell clusters within 8 h after cell culturing on the hydrogels, with an average size of ~ 300–400 μm (Figure 6a–e). A continuous increase in spheroid diameter was observed with time (Figure 6a–h). Most cells were arranged in cell islands or loose cell aggregates. HeLa spheroids with a mean size of ~700–800 μm were observed, and only low numbers of non-adherent cells were observed in the growth medium. The 3D structure shown by the spheroids was characterized by being compact and having a round shape at all times (Figure 6a–h). In general, a homogeneous pattern of spheroid size developed. The characteristic 3D structure was established after 72 h in culture for subsequent analyses.

On the other hand, PVA is a hydrophilic polymer that inhibits the deposition of serum proteins, which promote cell–substrate attachment and proliferation, favoring cell–cell interactions and facilitating the formation of spheroids [12,48]. The formation of cellular aggregates was demonstrated when the surfaces of the plastic dishes were coated with PVA aqueous suspensions, favoring 3D cell culturing [12]. Cellular aggregates of glioma cell lines and human embryonic kidney cells were formed on the third day of culture, showing large cell aggregates on the PVA-coated plates [12].

Hydrogel stiffness had a significant effect on cell function. Soft substrates may be advantageous in enhancing cell function. Due to spheroid morphology improvement, agarose hydrogels at 2% wt were optimal for spheroid formation [49].

Moreover, variations in hydrogel stiffness, density, composition, orientation, and viscoelastic characteristics affect cell activity and phenotype [52]. This highlights that the hydrogel’s composition, stiffness [44,46], and topography are essential in forming compact cell clusters, conserving their 3D structure and spherical shape [10,12,47].

### 2.5. Histological and Immunohistochemical Analysis

Cells cultured in 3D Petri Dish^®^ molds form cell clusters through the action of attractive adhesion and cohesion forces, surface adhesion molecules, and cytoskeletal elements [47]. Moreover, in 3D cell cultures, poor nutrient and waste transport lead to low stability, survival, and functionality over extended periods, presenting outstanding challenges [53]. We hypothesized that PVA hydrogels retain more culture medium in their 3D network than agarose hydrogels, favoring cell functions due to nutrient-controlled release to the cell culture according to the water absorption capacity.

**Figure 7:**
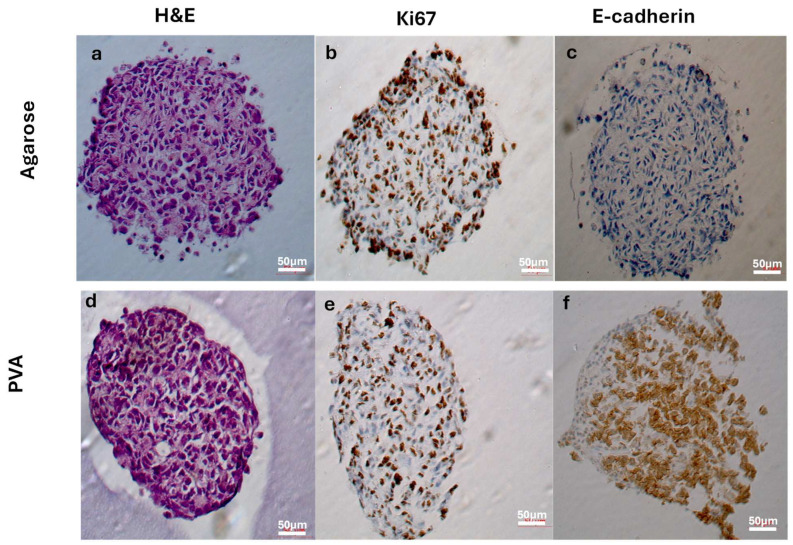
Histological and immunohistochemical analysis. HeLa cell spheroids after 72 h of culture in a standard medium in hydrogels. Histological images of H&E-stained HeLa spheroids after 72 h of culture (scale bar, 50 μm) in agarose (**a**) and PVA (**d**) hydrogels. Immunohistochemistry of HeLa spheroids in hydrogels after 72 h of culture (scale bar, 50 μm). Positive staining reactions for Ki67 are observed in HeLa spheroids cultured from agarose (**b**) and PVA (**e**) hydrogels with no difference in the staining pattern. No E-cadherin expression was observed in HeLa spheroids from agarose hydrogels (**c**), but a positive staining reaction was observed in HeLa spheroids from PVA hydrogels (**f**).

The results of the histochemical and immunohistochemical staining are shown in Figure 7. Histological staining was performed with hematoxylin and eosin (H&E) for general evaluation of the cell cultures [54]. H&E-stained 3D cultivated cells (72 h) exhibited characteristic tumor cell nuclei. Epithelial-like cells were densely arranged in the inner area and partly flattened at the periphery of the spheroids formed in the agarose and PVA hydrogels (Figure 7a,d).

We applied immunohistochemistry to further characterize the spheroids. Proliferative activity was assessed by anti-Ki-67 staining and E-cadherin was used for evaluating cell–cell interactions. The Ki-67 protein is strictly associated with cell proliferation and is expressed during all cell cycle phases except G0 [55]. This protein is used as a cell proliferation marker in pathology and a Ki-67 labeling index is employed for the diagnosis and prognosis of cancers [55,56].

The agarose hydrogel spheroids showed Ki-67 (brown) expression predominantly at the periphery, although well-distributed expression was also seen in the center (Figure 7b). Meanwhile, Ki67 expression at the periphery was minimal in the PVA hydrogel spheroids (Figure 7e). The results suggest that cell growth and proliferation are present in both samples and are more evident in the spheroids from agarose. In Hela spheroids generated by the hanging-drop method, the cell proliferation (Ki-67) staining patterns were not homogenously distributed in spheroids on day 12 of the culture [57].

E-cadherin (brown) was expressed only in the PVA hydrogel spheroids (Figure 7c,f). These findings indicate that PVA stimulates the expression of surface proteins such as E-cadherin. Cells settled at the bottom of the hydrogel wells were channeled together due to the concave design of the well bottoms, maximizing cell–cell interactions in the spheroids from PVA hydrogels. E-cadherin is a 120 kDa calcium-dependent transmembrane glycoprotein involved in intercellular interactions, epithelial tissue polarity and integrity [58], establishing cell–cell and cell–ECM interactions and is thus suitable for visualizing the formation of the ECM within the spheroids [13,54,59,60]. Epithelial cells in solid tumors typically adhere to each other using adhesion proteins such as E-cadherin to form solid structures [59]. A loss of E-cadherin expression has been reported as a hallmark in epithelial–mesenchymal transition (EMT), which plays an important role in tumor development, invasion, and metastasis [61]. In cervical epithelium cells, E-cadherin exhibits high expression along the cell membrane, mediating cell–cell adhesion. However, studies have shown that a loss of membranous E-cadherin expression has been reported in the cervical cancer HeLa cell line, as illustrated in Figure 7c [62,63]. Interestingly, our findings demonstrate an upregulation of E-cadherin expression in PVA hydrogels, but not in agarose hydrogels (Figure 7f). This observation aligns with reports suggesting that E-cadherin expression can be retained or upregulated in invasive carcinomas [64,65].

In 3D cultures, strong E-cadherin expression centered mainly around the spheroid nucleus due to the high cell density and cell–cell interactions being favored [66]. The effects of PVA on human pancreatic ductal adenocarcinoma (PDAC) cell lines were evaluated in serum-free 2D and 3D cultures, demonstrating that PVA induces alterations similar to the mesenchymal-to-epithelial transition, including increased E-cadherin and decreased Vimentin and N-cadherin expression [48].

HeLa cells seeded in an alginate-based matrix fabricated by 3D bioprinting showed high cell viability and spheroid formation until 6 weeks, reaching diameter dimensions of several hundreds of microns. Interestingly, the 3D bioprinted microenvironment favors the expression of E-cadherin within the 3D HeLa spheroids [60].

### 2.6. Anticancer Drug Sensitivity Tests

In order to unravel the impact of anticancer drugs on cell junctions in 3D culture, HeLa spheroids were treated after 24 and 72 h of cell culture with cisplatin (Cis-diamminedichloridoplatinum or Cis), a well-known alkylating agent with anticancer properties. The Cis causes DNA damage and cell cycle arrest by binding to guanine nucleotide bases and blocks DNA replication [67].

**Figure 8:**
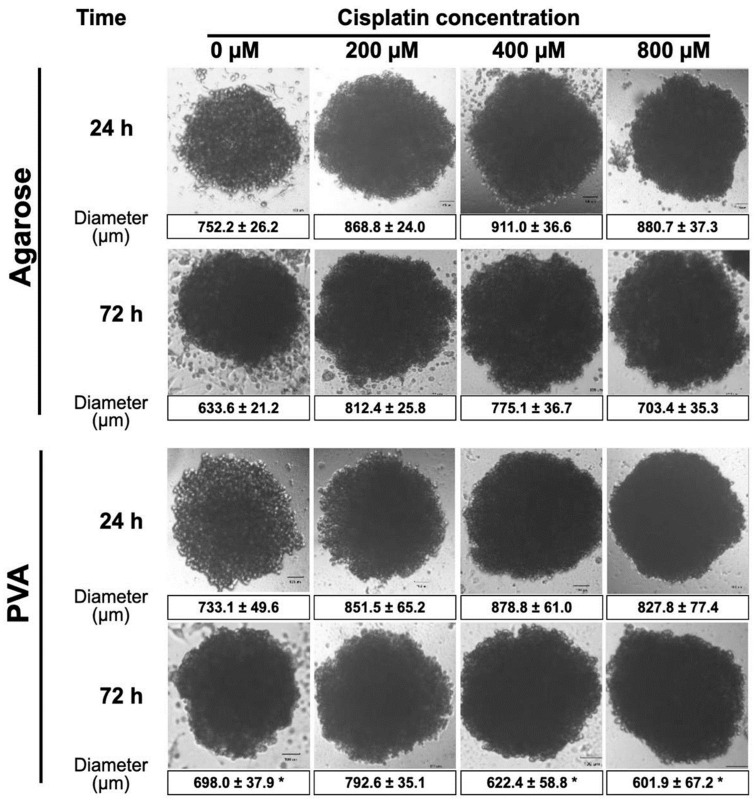
Anticancer drug testing. HeLa spheroids are exposed to different cisplatin concentrations after 24 and 72 h in a standard medium containing 200, 400, and 800 µM Cis. The spheroids were previously formed in agarose and PVA hydrogels (scale bar 100 μm). The values are expressed as the mean ± standard deviation (SD) of three independent experiments. The data were analyzed with a two-way ANOVA followed by Tukey´s multiple comparison test. * *p* ≤ 0.05 comparing agarose to PVA at the same time during treatment.

The drug was administered in different concentrations (200, 400, and 800 µM) to spheroids from both hydrogels. Figure 8 shows the results of the size and morphology of spheroids after Cis exposure using an inverted microscope. The size of the spheroids from the agarose hydrogels increased when the Cis concentration increased regardless of exposure time. The size of the spheroids increased from ~725 μm to ~911 μm at 24 h. Meanwhile, a size increase was observed from ~633.31 μm to ~775.12 μm at 72 h.

Similar behavior was observed in the spheroids from the PVA hydrogels at 24 h regardless of Cis concentration (from ~733.12 μm to ~851.53 μm) and at 72 h only for the lower concentration (from ~ 698 μm to ~792.56 μm). However, at 72 h, at concentrations of 400 and 800 mM, a decrease in size was observed from ~698 μm to ~601.92 μm.

Similar results were observed when Hela spheroids were exposed to 20,40, 80, 160, and 320 µM of Cis, showing that Hela spheroids maintain their shape without morphological changes, suggesting they might be insensitive to the drug [67].

Cell viability was investigated by live/dead staining (calcein/ethidium homodimer) after the spheroids were exposed to different concentrations of Cis and cultured for 72 h. It was crucial to know whether the integrity of the spheroids remained intact after exposure to cisplatin. The reason for using this stain is to observe the integrity of the plasma membrane. Calcein-AM (green staining) indicates intracellular esterase activity, whereas internalized ethidium homodimer (red staining) represents the loss of cell membrane integrity [68].

**Figure 9:**
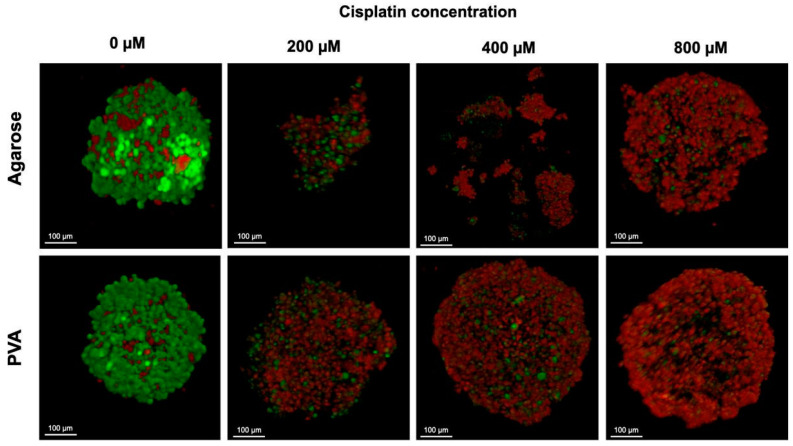
Confocal microscopy image of live/dead staining. HeLa spheroids after 72 h of cisplatin treatment. Live and dead cells were stained green and red, respectively (scale bar 100 μm).

Figure 9 shows the images of the live/dead stained spheroids from the agarose and PVA hydrogels. Furthermore, the spheroids formed in the PVA hydrogels were more compact than those from the agarose hydrogels. The macroscopic appearance of spheroids without Cis exposure showed a spherical structure. Most cells were alive (green signal), and some dead cells were observed (red signal) inside the spheroid. The results indicate high cell viability, suggesting an adequate exchange of nutrients and gases, and therefore, the growth and functionality of HeLa cells. Treated spheroids from the agarose hydrogels lost their spherical shape and appeared disintegrated. Some cells were separated from the main spheroid body or exhibited condensed nuclei due to cell death. Treated spheroids from the PVA hydrogels were spherical and looked more compact than those from the agarose hydrogels. No spheroid disintegration was observed regardless of the concentration of Cis to which they were exposed. The exchange of nutrients and gases and the growth and functionalities are also meaningful [14]. Three-dimensional spheroids have an external proliferating zone and an internal quiescent zone caused by the lack of nutrients and gas exchange (e.g., O_2_, CO_2_) [69].

## 3. Conclusions

PVA and agarose hydrogels prepared using the 3D Petri Dish^®^ micro-molds with a circular topography can be used to form HeLa spheroids for potential use in developing anticancer drugs. The agarose hydrogels were obtained through gelation of an agarose suspension at a temperature of 37 °C. Meanwhile, the PVA hydrogels went through a cryogenic process based on a cycle of freezing/thawing from a PVA suspension. The cryogenic process favored the physical cross-linking of the PVA, demonstrated by the FTIR measurements. Hydrogels can easily be manipulated manually. The agarose hydrogels were significantly more rigid than the PVA hydrogels. The PVA hydrogels have a better water uptake capacity than the agarose hydrogels, which allows for better diffusion of oxygen and nutrients. The cell spheroids can be harvested by rinsing them with a culture medium, facilitated by the low adhesion of the cells to the agarose and PVA surface, which favors cell–cell interactions. The cells do not adhere to the agarose or PVA hydrogels, and they spontaneously self-assemble through cell–cell binding into shapes that conform to the recesses. The size of cell spheroids can be controlled by the number of cells seeded in the well or by the culture time using agarose and PVA hydrogels.

The generated spheroids in PVA showed the expression of E-cadherin and greater integrity than in agarose, which is very useful for drug testing. In this regard, our findings suggest that the surface chemistry of micro-molds could influence cell behavior and spheroid formation. However, more detailed studies are required to support this hypothesis. The results demonstrate that HeLa spheroids formed in PVA hydrogels under our experimental conditions are a more suitable option for in vitro anticancer drug screening since they remain intact.

## 4. Materials and Methods

Poly (vinyl alcohol) [PVA (molecular weight ~89 kDa and hydrolysis degree ~99.8%)] and agarose of low gelling temperatures were purchased from Sigma Aldrich (St. Louis, MO, USA). All other solvents and reagents were of analytical grade.

For the cell culture, Dulbecco’s Modified Eagle’s Medium high-glucose medium (DMEM high-glucose medium, Sigma Aldrich) and Fetal Bovine Serum (Gibco, Grand Island, NY, USA) were used. For the viability assay, a Live/Dead™ Cell Imaging Kit was used (Catalog Number: R37601).

### 4.1. Hydrogel Preparation

The 3D Petri Dish^®^ micro-molds used for the patterned hydrogels were the 12–81 large spheroids (MicroTissues, Inc., Sharon, MA, USA). This micro-mold has 81 circular recesses (9 × 9 array). The nominal dimensions of one of these recesses when formed in the 3D Petri Dish^®^ are a diameter of 800 μm and a depth of 800 μm. The single large cell seeding chamber above these 81 recesses holds a volume of 190 μL. This 3D Petri Dish^®^ fits into a standard 12-well tissue culture plate (6 micro-molds per pack). The micro-molds were previously washed in distilled water and ethanol 70 wt% (3 times) and irradiated using ultraviolet radiation at 254 nm at 120,000 μJ/cm2 for 30 min in a CL-1000 UV Crosslinker (Cambridge, UK) before use.

#### 4.1.1. Poly(vinyl alcohol) Hydrogels

A PVA suspension (1% wt) was prepared by dissolving PVA powder in distilled water and gently stirring it for 4 h at 90 °C. After that, 0.5 g of polymer suspension was transferred into 3D Petri Dish ^®^ micro-molds and frozen–thawed at −20 °C for 24 h with subsequent thawing (1 cycle).

#### 4.1.2. Agarose Hydrogels

An agarose suspension (2% wt) was prepared by dissolving agarose powder in distilled water and gently stirring it for 2 h at 37 °C. After that, it was sterilized by autoclaving. The agarose suspension (0.5 g) was transferred into the micro-molds and cooled at 4 °C for 15 min until the agarose gelled.

### 4.2. Hydrogel Characterization

#### 4.2.1. Physical Appearance

The hydrogels were inspected visually for their color, appearance, uniformity, size, and weight. The hydrogels’ size was measured using a vernier caliper (Precise, ToolUSA, Las Vegas, NV, USA). The hydrogels were weighed using an analytical balance (Cobos ATX224, Barcelona, Spain). All measurements were performed in triplicate.

#### 4.2.2. Macroscopic Morphology

The hydrogels were examined by stereoscopic microscopy (Nikon C-LEDS Hybrid LED Stand, Tokyo, Japan) with an adapted camera (INFINITY1-3C) for macroscopic morphology analysis.

#### 4.2.3. Fourier Transform Infrared Spectroscopy

Analysis by FTIR was performed on dried hydrogels and films (prepared by using the casting solvent technique) [70]. The samples were dried in a convection oven (Luzeren, Hangzhou, China) at 37 °C for 24 h. The dried samples were placed on the ATR (attenuated total reflectance) surface of the Spectrum GX Spectra (Perkin Elmer, Waltham, MA, USA). Spectra were obtained from 4000 to 400 cm^−1^. Spectra were obtained with 24 scans and 4 cm^−1^ resolution and normalized with an ordinate limit of up to 1.0 absorbance using the tool available in the spectrometer software (Spectrum, v5.01, Perkin-Elmer, 2003).

#### 4.2.4. Texture Analysis

The texture profile analysis (TPA) was performed using a texturometer, TA-XT PLUS (Stable Micro System Co., Ltd., Surrey, UK), through two consecutive compression cycles of the samples using a cylindrical probe of 50 mm in diameter. The test conditions were as follows: test speed of 0.5 mm s^−1^, trigger point of 0.05 N, strain of 45%, strain time of 5 s, and charge cell of 5 kg. The textural parameters were hardness, gumminess, chewiness, cohesiveness, springiness, and elasticity (Young’s modulus). Ten measurements were performed, and the average ± standard deviation was reported.

Young’s modulus is obtained by fitting the linear part of the force–displacement applied by the specimen with a linear equation. The slope of this equation corresponds to Young’s modulus or the stiffness. Stiff materials have high moduli and vice versa.

#### 4.2.5. Swelling Studies

The percentage of swelling was measured using the following methodology, which consisted of taking at different times (0, 5, 10, 20, 40, 60, 80, and 120 min) the weights of the hydrogels of agarose and PVA with DMEM high-glucose medium and comparing the weights at different times against the initial weight, using the following formula:

$$Swelling(\%)=\frac{Wt-W_{0}}{W_{0}}\times 100$$

where:

*Wt* = weight of hydrogel at different times;

*W*_0_ = initial weight

All measurements were performed in triplicate.

### 4.3. Spheroid Formation

#### Cell Culture

DMEM culture medium supplemented with 10% (*v*/*v*) of heat-inactivated FBS and 1% (*v*/*v*) of a double antibiotic (100 mg/mL of streptomycin and 100 U/mL of penicillin) was used to maintain the HeLa cell line. The HeLa cells were grown in an incubator at 37 °C with 90% humidity and 5% CO_2_. Subsequently, the cells were subcultured thrice a week until 80% cell confluence was obtained. The cells were detached with trypsin (0.25%), and the cell count was carried out using the blue trypan technique.

The PVA and agarose hydrogels were carefully removed from the 3D Petri Dish^®^ micro-molds without breaking them, poured into a cell culture plate (12-well), followed by phosphate buffer (3X, PBS) and ethanol (3X, 70% *v*/*v*) washes, and finally exposed to ultraviolet (UV) light for 1 h inside the safety cabinet class 2 (Purifier Logic 4′-Labconco) for the sanitization process and disinfection, and the culture medium was removed. To equilibrate the PVA and agarose hydrogels, a culture medium (DMEM high-glucose medium) with antibiotic penicillin/streptomycin (1% p/s) was added (1 mL/well) and incubated at 37 °C with 90% humidity and 5% CO_2_ for 1 h, and the culture medium was removed.

After that, HeLa cells were seeded at a density of 3.5 × 10^6^ cells per well (200 µL) and cultured at 37 °C with 90% humidity and 5% CO_2_ for 20 min. Then, an additional medium was added to the outside of the hydrogels (1 mL of DMEM high-glucose medium, 10% FBS, and 1% p/s antibiotic) and incubated at 37 °C with 90% humidity and 5% CO_2_ for 72 h for spheroid generation. The surrounding exchange medium was removed every 24 h (Casting, Equilibrating, and Seeding of the 3D Petri Dish^®^ protocol by Microtissue Inc. was followed).

After 48 h of culture, the medium was removed, and the spheroids were recovered. The hydrogels were carefully removed and inverted under the microscope to ensure the spheroids were released and transferred to a new 12-well plate with 1 mL of culture medium. Afterward, the covered culture well plates were centrifugated at 500 rpm/5 min (Centrifuge Eppendorf 5430/5430 R). The hydrogels were discarded, and the spheroids remained on the culture plate for in vitro assessment

### 4.4. In Vitro Spheroid Assessment

#### 4.4.1. Measurement of Spheroid Size

Spheroids formed with HeLa cells were imaged using an inverted microscope, Optika (XDS-2FL), and the images were analyzed using Infinity Analyze and Capture Windows v6.5.6 software. Briefly, all images were converted to simplified threshold images under the same converting condition, and the edges of the spheroids were detected using a selection tool. The diameters of the detected spheroids were initially measured as pixels and converted to micrometers.

#### 4.4.2. Histochemistry and Immunohistochemistry

Sample preparation: HeLa spheroids were rinsed in PBS and immediately fixed with 10% neutral buffered formalin (in PBS) at room temperature for 24 h, and formalin was carefully aspirated. The samples were then rinsed in PBS again and embedded in 2% wt agarose (Sigma Aldrich). The excess agarose was removed with a scalpel, and the agarose blocks containing the samples were processed in an Automatic Benchtop Tissue Processor (Leica TP1020). The dehydration process was carried out by subjecting them to two vats of formalin for 1.15 min/each and two subsequent vats of 96% alcohol for 1:15 min, followed by two vats of absolute alcohol for 1:15 min each. The clarification step consisted of immersing them in two tubs of xylol for 1:15 min/each, and finally, the inclusion was started by subjecting them to two tubs of paraffin for 1:15 min/each. Subsequently, the agarose blocks were removed from the capsule and included in the paraffin block embedder (Myr EC350-1). Once the blocks were obtained, they were placed on an ice plate. The blocks were cut on a microtome (Leica Model RM2125RTS) at 3 microns and allowed to float in a flotation vat at 50 °C (Leica model HI1210), and then mounted on slides, and finally, they were dewaxed in a Felisa oven at 70 °C for 15 min.

Histological staining was performed with hematoxylin and eosin (H&E) for architectural and morphology analysis. First, the slides were placed in a Harris Hematoxylin solution for 5 min, followed by two baths in 1% acidic alcohol, rinsed with running water, and placed in ammonia water for 30 s. They were then washed with running water and immersed in a 96% alcohol bath containing eosin for 1 min. Five washes were carried out with running water. For dehydration, ten alcohol washes were made in two ethanolic baths (one at ethanol at 96% and the other in absolute ethanol), followed by xylol washes. Samples were observed under fluorescence microscopy (Iroscope, IRO-MG-321FL, Sonora, México).

Immunohistochemistry was carried out on 3 μm sections of paraffin-embedded samples. Afterward, the samples were mounted on electrocharged slides (Biocare Medical, Pacheco, CA, USA) and dewaxed in a Felisa oven at 70 °C for 2 h. Immunohistochemistry was performed on semi-automatic BenchMark ULTRA equipment in a Ventana Roche Discovery Immunostainer (Mannheim, Germany) using a DAB-MAP discovery research standard procedure. The mounted sections were incubated with the appropriate primary antibody at 37 °C for 1 h. The specimens were then incubated with Discovery Universal Secondary Antibody, Ventana 790-4497 (E-cadherin, 100 µL), and Ventana 760–124 (Ki67, 100 µL) for 30 min at room temperature. Detection was achieved using the DAB-MAP Detection Kit (Ventana) according to the diaminobenzidine (DAB) development method. Antibody detection was attained with the DAB-MAP Detection Kit (Ventana 760–124) using a combinatorial approach involving the diaminobenzidine development method with copper enhancement followed by light counterstaining with hematoxylin (Ventana 760–2021) for 4 min. The stained sections were then manually dehydrated using an upgraded alcohol series, clarified with xylene, and then permanently mounted with Entellan (Merck, Darmstadt, Germany). Samples were observed under fluorescence microscopy (Iroscope, IRO-MG-321FL, Sonora, México).

### 4.5. Anticancer Drug Sensitivity Tests

The anticancer drug sensitivity of HeLa cells in a monolayer was evaluated by determining the 50% lethal dose (DL_50_) of cisplatin (PiSA Farmacéutica, Guadalajara, México) using an MTT assay (3-(4,5-dimethyl thiazolyl-2) 2,5-diphenyltetrazolium bromide salts). HeLa cells were cultured in a monolayer using high-glucose DMEM culture medium supplemented with 10% FBS (*v*/*v*) and 1% antibiotics and incubated at 37 °C with 90% humidity and 5% CO_2_ after 48 h of cisplatin (PiSA Farmaéutica) exposure at different concentrations (19.9 µM, 41.5 µM, 83 µM, 170 µM, and 330 µM).

The cisplatin sensitivity of Hela spheroids formed in the PVA, and agarose hydrogels was evaluated by microscopic visualization and cell viability determination. The spheroids were exposed to cisplatin at 200, 400, and 800 µM for 24 and 72 h. The spheroids were monitored in terms of size and integrity by images captured by an inverted microscope (Optika, XDS-2FL/Infinity Analyze Capture Windows v6.5.6 software). Three independent experiments were conducted. ImageJ software (version 1.52a, Wayne Rasband, National Institutes of Health, Bethesda, MD, USA) was used for size measurements of the spheroids.

HeLa cell viability within the spheroids was evaluated with a modified protocol from the Live/Dead^®^ viability/cytotoxicity kit (Molecular Probes™ Invitrogen, Carlsbad, CA, USA). Cell viability was determined using a confocal microscope (Leica DM550Q, Wetzlar, Germany) and ImageJ software (version 1.52a, Wayne Rasband, National Institutes of Health, Bethesda, MD, USA). The live/dead assay differentially labels live and dead cells with fluorescent dyes. Live cells are stained green to indicate intracellular esterase activity with calcein-AM (Live Green), while dead cells are stained red to indicate a loss of plasma membrane integrity with ethidium homodimer-1 (Dead Red).

### 4.6. Statistical Analysis

The data were expressed as the means of triplicate samples ± standard deviations. The arithmetic media and % variation coefficient were used. A one-way analysis of variance was applied, and whenever appropriate, Tukey’s test was used to determine differences among the means (*p* < 0.05). Statistical analyses were performed using Statgraphics Centurion XVI program version 16.1.17 (StatPoint Technologies, Inc., Warrenton, WV, USA).

## Data Availability

The raw data supporting the conclusions of this article will be made available by the corresponding authors upon request.
